# Monitoring and Evaluating Progress towards Universal Health Coverage in China

**DOI:** 10.1371/journal.pmed.1001694

**Published:** 2014-09-22

**Authors:** Qingyue Meng, Ling Xu

**Affiliations:** 1 China Center for Health Development Studies, Peking University, Beijing, China; 2 Center for Health Statistics and Information, China National Health and Family Planning Commission, Beijing, China

## Abstract

This paper is a country case study for the Universal Health Coverage Collection, organized by WHO. Qingyue Meng and colleagues illustrate progress towards UHC and its monitoring and evaluation in China.

*Please see later in the article for the Editors' Summary*

This paper is part of the PLOS Universal Health Coverage Collection. This is the summary of the China country case study. The full paper is available as Supporting Information file Text S1.

## Background

China is the most populated country in the world and one of the countries with the fastest economic growth over the past three decades. Inequity in health has arisen as a large concern for Chinese society. A new round of health system reforms was initiated by the government in early 2009, aiming to establish a health system in which all people can access basic health care through an equitable, efficient, affordable, and effective health system [1], which coincides strongly with the basic concept of universal health coverage (UHC) defined by World Health Organization [2].

## Universal Health Coverage: The Policy Context

The reforms initiated in 2009 have focused on improving social health insurance schemes in both rural and urban areas, strengthening the primary health care system, supporting delivery of essential public health programs, removing drug markups from the financing of the primary health providers, and reforming the public hospital sector. All these reform areas are closely linked with improving access to affordable and quality health care for all. Prior to the reforms, China had begun expanding health insurance schemes for the rural population in 2003 and for urban unemployed individuals in 2007.

## Monitoring and Evaluation for UHC

The principal source of information for UHC monitoring and evaluation is the two main databases managed by the Center for Health Statistics and Information of the National Health and Family Planning Commission. One is the national health services survey database, which provides individual-based data on access, health care utilization, and medical expenditures. The other database contains the routine reporting data of health facilities, which provides basic information on public health providers. Information for analyzing contextual factors for UHC including economic development, population, the social security system, and education can be obtained from the China Statistical Year Books that are produced by the National Bureau of Statistics.

Indicators for monitoring and evaluating UHC in China can be placed into three categories of institutional, service, and cost coverage. Institutional coverage refers to the institutionalized mechanisms and programs that include financial protection mechanisms (health insurance schemes), provision of essential public health programs, and mechanisms for mobilizing human resources and improving the quality of health care.

## Progress towards UHC in China

Using data from the National Health Services Surveys [3], service and financial coverage are analyzed with selected indicators.

Population coverage in the antenatal program, an indicator of maternal and child health care, has reached a high level (95% in 2008); however, the disparity in the concentration of services was still great between rural and urban areas in 2008, with urban pregnant woman receiving 8.3 visits versus 4.7 visits for rural pregnant women. Levels of smoking and blood pressure tests per year are used as measures to reflect the delivery and effectiveness of health promotion interventions and services targeting non-communicable diseases. Smoking rates declined between 1993 and 2008 in both urban and rural areas, but remained at high levels in 2008. The blood pressure testing rate, a newly added indicator in the national health services survey in 2008, was at less than 50% of adults in 2008. Cure rates for tuberculosis, which is used to measure service coverage of infectious diseases, declined between 2003 and 2008 in both urban and rural areas. Unmet health needs that are used for measuring access and health care utilization of curative care services are higher in rural than in urban areas. In 2008, of the total number of people with unmet inpatient care because of financial hardship, 71.4% were located in rural areas and 67.5% in urban areas.

**Figure 1:**
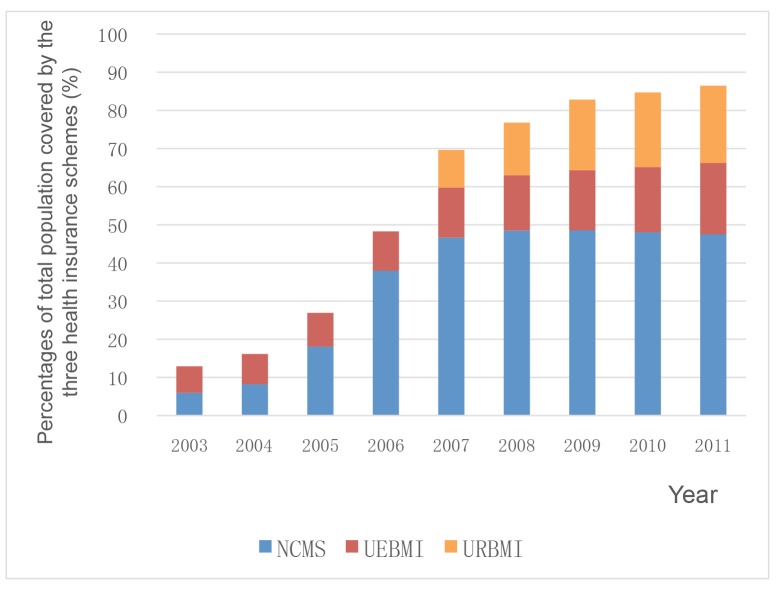
Population coverage by the three health insurance schemes, 2003–2011. Data sources: [4]–[6].

**Figure 2:**
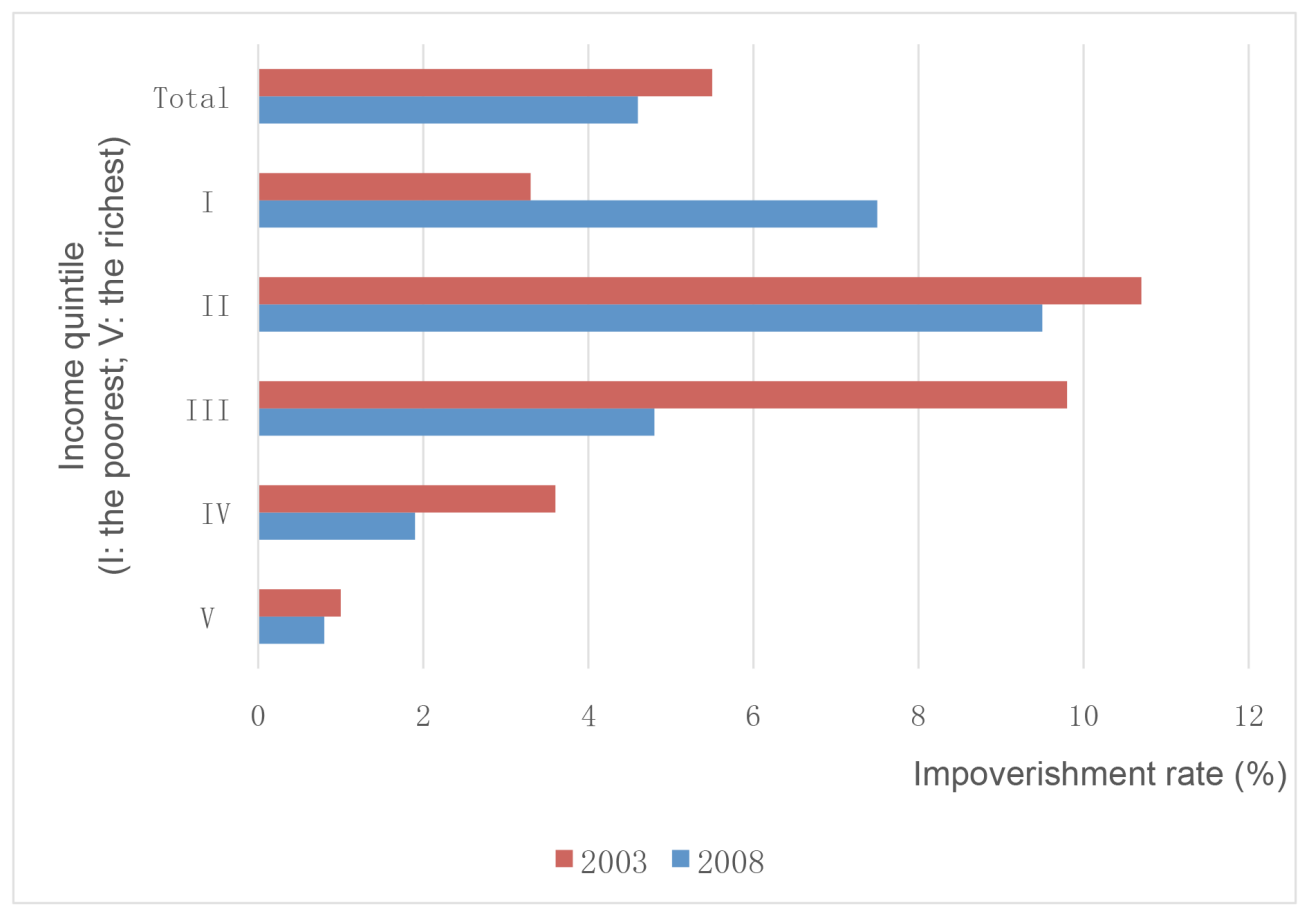
Percentage of households below the poverty line due to medical expenditures (%). Data source: [3].

Establishing prepayment systems has been a key strategy in China to provide people with financial protection. Figure 1 shows the population coverage of the three schemes from 2003 to 2011, with a rapid expansion from the mid 2000s [4]–[6]. Between 2003 and 2008, the impoverishment rates decreased by 0.9%, but the poorest income group increased dramatically mainly owing to a rapid increase in health care utilization and a relatively low capacity of protection mechanisms for these individuals (Figure 2) [3].

## Conclusions and Recommendations

Analysis of the progress towards UHC with selected indicators shows positive trends, especially in service coverage. In terms of financial protection, nearly all Chinese people have been covered by either rural or urban health insurance schemes. Although the impoverishment rate continued to increase for the poor between 2003 and 2008, the overall impoverishment rate and proportion of out-of-pocket payments in total health expenditures have declined.

A number of challenges must be addressed to accelerate UHC in China. First and second, both equity in and the quality of health care need continuous improvement. Third, the cost escalation of medical care should be appropriately contained. Finally, the concept of Health in All policies (the inclusion of health considerations in policy making across different sectors that influence health) needs to be operationalized. Major recommendations include making the health financing system more equitable, strengthening the primary health care system, and keeping cost escalation of medical care under control.

## Supporting Information

Text S1 **The full country case study for China.**
(DOCX)

## Abbreviations

UHC: universal health coverage
