# Supplementary material for: Monitoring and Evaluating Progress towards Universal Health Coverage in China
Source: PLoS Med. 2014 Sep 22;11(9):e1001694. doi: 10.1371/journal.pmed.1001694 (PMC4170954; doi:10.1371/journal.pmed.1001694)
Supplement: Text S1 — The full country case study for China. (DOCX) [file pmed.1001694.s001.docx]

**Full Case Study: Monitoring and evaluating progress towards Universal Health Coverage in China**

Qingyue Meng^1^ and Ling Xu^2^

^1^ China Center for Health Development Studies, Peking University, Beijing, China

^2^ Center for Health Statistics and Information, China National Health and Family Planning Commission, Beijing, China

**This paper is the full country case study to accompany the summary paper “Monitoring and evaluating progress towards Universal Health Coverage in China” that is part of the Universal Health Coverage Collection. Not commissioned; externally reviewed.**

**Abstract:** China is the most populated country in the world and one of the countries with fastest economic growth over the past three decades. Chinese population health has greatly improved over the past six decades, mainly contributing to primary health care focus and availability of financial protection mechanisms. However, the country has also experienced a decline in the level of health system performance. A new round of health systems reform was initiated by the government in early 2009, aiming to provide accessible and high quality health care to all people that is consistent with the concept of universal health coverage (UHC). For monitoring and evaluation of progress towards UHC, indicators focusing on service and cost coverage have been developed and analyzed using the databases available. The analysis shows that UHC in China has made good progress in selected indicators, especially on service coverage. However, the gaps in service coverage between urban and rural area, regions, and income quintiles exist. Overall proportion of out-of-pocket in total health expenditures has declined, but the impoverishment rate continued to increase for the poor between 2003 and 2008. Inequity in health is one of the biggest challenges in achieving UHC. To establish a more equitable health financing system and to strengthen the primary health care delivery system are the key strategies towards UHC.

**Summary Points:**

1. Inequity in health is one of the biggest challenges in China for achieving a more equitable society. Universal health coverage is aim of the current health system reform.

2. Information systems including national services survey and health account studies are available in monitoring and evaluation towards UHC.

3. China has made good progress in improving both service coverage and cost coverage. Access to basic health care has been improved, especially for the poor. All people have been covered by health insurance schemes.

4. For achieving UHC, China needs to continue to improve equity in access to health care and to control cost escalation of medical care.

**1. Background**

China is the most populated country in the world with 1.34 billion people accounting for 19% of the total global population by end of 2010 [1]. Over the past three decades, the size of urban residents has been rapidly expanded as a result of rural migration. In 2011, there were 230 million migrants which accounted for 17% of the total population [2]; 9.1% of the total population was at and above 65 years old [3].

The last three decades have seen a miracle in economic development in China with an average of 9.9% annual economic growth rate between 1978 and 2010[4]. In 2011, China entered into the middle-income country group with per capita GDP (gross domestic product) 8,400 US$ (PPP, purchasing power parity) [4]. While the economic situation is greatly improved, gaps in per capita wealth between urban and rural, regions and population groups have widened. The ratios of per capita income of urban resident over rural residents increased from 2.5 in 1980 to 3.13 in 2011 [5].

Chinese population health has been greatly improved over the past six decades. From 1980 to 2010, the life expectance years increased from 67 years old to 73.5 years old, and infant mortality declined from 47.9 to 13.1 per thousand live births [3, 6]. Disparities in health status exist between the urban and rural areas, regions, and population groups. In 2010, infant mortality in rural area was 2.8 times higher than that in urban area, maternal mortality ratio in rural area was 2.2 times higher than that in urban area [3]. Non-communicable diseases have been the major health threats in China].

Performance of health systems varied in different development stages in China. From 1950s to 1970s when China was a low-income country, investing in basic health infrastructures, training primary health workers, expanding coverage of basic financial protection mechanisms, and coordinating health-related sectors were the major policies and actions from the government in making a health system function [7]. However, China also experienced a decline in the level of health system performance between 1980s and 1990s. For example, collapse of rural cooperative medical scheme in mid 1980s led to a rapid increase in the number of households who incurred catastrophic medical expenditures [7]; financing of public hospitals relying on user fees led to cost escalation of medical care and increasing unmet health needs of the poor [8]; and inappropriate incentives for health providers led to concentration of qualified health workers in urban and tertiary hospital settings, which constrained access to quality health care for the poor and people living in rural and remote area. Since early 2000s, Chinese government has started to develop and implement a series of policies to strengthening its health systems.

The concept of the universal health coverage (UHC) was clearly defined in the aim of health system reform that was initiated by the central government in early 2009. The aim of the reform is to establish a health system in which all people can access to basic health care through an equitable, efficient, affordable and effective health system [9], which highly coincides with the basic concept of UHC defined by World Health Organization (WHO) [10]. UHC has been increasingly used by both policy makers and academia in China for assessing progress of the health systems reform. For example, the concept of coverage in three dimensions (population, service, and cost) developed by WHO has been used by Chinese researchers in evaluating to what extent China has achieved UHC [11,12].

**2. Universal health coverage: the policy context**

The current health system reform aiming to achieve UHC in China has proposed and implemented a number of strategies, policies, and actions, mainly focusing on health systems financing, health care delivery system, human resources in health, and essential medicines policy. Responsibilities of each level government in carrying out the reform activities are defined and evaluated periodically. Over the past few years, the reform has focused on improving the social health insurance schemes in both rural and urban areas, strengthening primary health care system, supporting delivery of essential public health programs, removing drug markups from finance of the public health providers, and reforming the public hospital sector. All those actions are closely linked with improvement of access to affordable and quality health care by all people.

Before this round of health system reform starting from 2009, China had already started expansion of financial protection mechanisms in health from early 2000s. In 2002, the central government decided to establish a new rural cooperative medical scheme (NCMS) targeting the entire rural population [13]; and in 2006, the central government decided to support a health insurance scheme for all urban residents outside formal economic sector [14]. Those two health insurance schemes provide a prepayment mechanism for majority of the Chinese people in use of basic medical care services.

**3. Monitoring and evaluation for UHC**

This section introduces information systems available and indicators developed for UHC monitoring and evaluation.

**3.1 Information systems and indicators for UHC monitoring and evaluation**

The main information source available is in the Center for Health Statistics and Information of the National Health and Family Planning Commission (NHFPC). This center manages two main databases, the national health services survey databases which provides comprehensive information on access, health care utilization, and medical expenditures by individuals, and routine reporting data of health facilities which provide basic information on public health providers. Other departments within NHFPC also organize information systems on specific topics. For example, Department of Rural Health Management of NHFPC is managing the information system on the rural cooperative medical scheme; Department of Diseases Control of NHFPC is managing reporting system of infectious diseases through China Center for Disease Prevention and Control; and Department of Maternal and Child Health of NHPFC is managing the reporting system for maternal and child health.

Information system through the China Statistical Year Book produced by the State Bureau of Statistics provides contextual indicators for analyzing UHC implementation and achievements, in which indicators on economic development, population, education, social affairs, health, and other aspects are included.

Besides indicators that can reflect the service coverage and financial coverage, indicators for reflecting coverage of institutionalized mechanisms and programs are emphasized in China in UHC. The institutionalized mechanisms and programs include financial protection mechanisms (health insurance schemes), provision of essential public health programs, and mechanisms for mobilizing human resources and improving quality of health care. Those mechanisms and programs are usually the entry pints for government to provide financial, administrative, and regulatory supports. Table S1 summarizes the major indicators that have been used for monitoring and evaluation for UHC in China. However, those indicators have not been formally standardized and are in process to be improved.

| **Dimensions of UHC** | **Categories of indicators** | **Major indicators** | **Data sources** |
| --- | --- | --- | --- |
| Institutional coverage | Financial protection mechanisms | Population coverage | HIS |
|  |  | Scope of health care | HIS |
|  |  | Cost sharing | HIS |
|  | Essential public health package | Availability of government subsidies | EPHP |
|  |  | Fulfillment rate of required health care delivery | EPHP |
|  | Primary care-focused delivery system | Availability of health infrastructures | Routine report |
|  |  | Availability of qualified health workers | Routine report |
| Service coverage | Maternal and child health | Antenatal visit | NHSS |
|  |  | Postnatal visit | NHSS |
|  |  | Institutional childbirth | NHSS |
|  |  | Screening of maternal diseases | NHSS, MCH |
|  |  | Child immunization | NHSS, MCH |
|  | Chronic conditions and injuries | Hypertension management | NHSS, EPHP |
|  |  | Diabetes management | NHSS, EPHP |
|  |  | Screening of cancers | NCD |
|  |  | Severe mental health case management | NHSS, EPHP |
|  |  | Smoking rate | NHSS, NCD |
|  | Infectious diseases | TB Detection rate | TB survey |
|  |  | Cure rate of TB | NHSS |
|  | General curative care | Health care utilization | NHSS |
|  |  | Physical access to health providers | NHSS |
|  |  | Unmet health care | NHSS |
|  |  | Distribution of health care utilization | NHSS |
|  | Safe water and sanitation | Safe drinking water | Statistical Yearbook |
|  |  | Sanitary toilet | Statistical Yearbook |
| Financial protection | Funding | Percentage of GDP for health | HAS |
|  |  | Government health budget | HAS |
|  | Financial burden | Out-of-pocket payment | HAS and NHSS |
|  |  | Catastrophic medical expenditure | NHSS |
|  |  | Impoverishment | HNSS |

**Table S1:** Indicators and source of information for UHC measurement and monitoring

**HIS:** Health insurance statistics system; **EPHP:** report on essential public health package system; **NHSS:** National health services survey; **MCH**: reports on maternal and child health; **NCD:** specific surveys and reporting systems on non-communicable diseases; **HAS:** Health account study.

**3.2 Introduction to selected information systems**

**3.2.1 The National Health Services Survey**

The National Health Services Survey (NHSS) is one of the most important sources of information on health status, health care utilization, health expenditures, and satisfaction of people with the health systems. NHSS is household-based survey starting from 1993 and has been organized every five years since then. Information from NHSS has been heavily used by both government and other organizations for analysis of health-related issues. From each of the surveys, a comprehensive analysis is publically reported and published in a book. NHSS also produce other types of products including articles and reports on specific topics.

Specific objectives of NHSS include 1) to assess health needs and health care utilization and their influencing factors; 2) to investigate population coverage of health security systems and to assess impacts of those systems on health care utilization and financial protections; 3) to analyze access of vulnerable population groups including children, maternal women, and the elderly to health care; and 4) to assess satisfaction of general public with health systems.

Households in the surveys are selected using a multiple stage cluster randomized sampling method. Rural counties and urban districts are fixed in all rounds of surveys, but households are randomly selected in those counties and districts in each of the surveys. The fifth NHSS is organized in 2013 with an enlarged sample size, about 300,000 individuals from 93,600 households in 156 rural counties and urban districts are investigated. Data collection of NHSS is conducted by investigators from provincial and county/district level institutions.

NHSS include indicators: a) social and economic status of households and individuals: demographic characteristics; income and expenditures; education; employment status; and occupations; b)health needs: self-report health status; prevalence of diseases and injuries; c)health care utilization: treatments of diseases; unmet needs and reasons; utilization of public health programs; and distributions of outpatient and inpatient care; d) coverage of social health security programs: coverage of health insurance schemes; coverage of medical assistance fund program; ; and cost sharing; and e) financial burden of diseases: out-of-pocket health expenditures; medical expenditures per outpatient visit; and medical expenditure per inpatient care.

**3.2.2 Health account study**

Health account study in China was initiated in early 1980s supported by the World Bank. In 1993, results of health account study at national level were officially released by National Health Economics Institute, which was endorsed by Ministry of Health (currently NHFPC). Since then, methodologies of health account studies have been developed by Chinese researchers with supports from international organizations, especially the World Health Organization and World Bank. In addition to national level study, health account studies are conducted at provincial level in about ten provinces. In 2002, China Statistic Yearbook started to publish the major results from health account study. Information from health account study has been the major source in decisions making of financial policies and analysis and monitoring of health expenditures.

Health account study publishes a report every year. The report includes following key information: a)total health expenditures: total health expenditures; and percentages of health expenditure in gross domestic products (GDP); b)source of health expenditures: health expenditures from government health budgets, social sector including social health insurance schemes, individuals; and historical analysis of shares of the above three sources; c)distribution of health expenditures: shares of health expenditures in hospitals, clinics, public health facilities, pharmacies, and other types of health providers; and d)analysis of health expenditures by regions and provinces: health expenditures by rural and urban areas; reports on health expenditure analysis by provinces where health account studies are conducted.

**4. Progress towards UHC in China**

This section report analysis of the progress towards UHC in China using selected indicators of service and financial coverage. We put health insurance scheme in financial coverage for its close linkage with financial protection.

**4.1 Service coverage**

Health services are provided by both health sector and non-health sector. After analysis of service coverage that are provided by health providers, safe drinking water is used as an example for service coverage by non-health sector.

**4.1.1 Coverage of health care services**

We analyze service coverage using data from the national health services surveys. The main results are presented in Table S2.

**Maternal and child health:** Coverage of antenatal care is a major indicator for maternal and child care. While the population coverage of antenatal program has reach high level (95% in 2008) [15], disparity in intensity of services were big between rural and urban areas. In 2008, antenatal visits were 8.3 times per pregnant woman of urban residents and 4.7 times for rural maternal women. Children’s vaccine immunization program is another important and frequently used indicator in maternal and child health. Immunization coverage of low-income group of children had been rapidly expanded between 2003 and 2008.

**Non-communicable diseases:** Hypertension and diabetes are mainly used as tracer conditions of NCDs in monitoring and evaluation of NCD control programs. Indicators including active finding rate of the diseases by the patients, case management rate, standard treatment rate and proportions of patients with complications are frequently used. Smoking rate and blood pressure test rate in a year are used to reflect delivery and effectiveness of health promotion interventions and services. Smoking rates declined between 1993 and 2008 in both urban and rural areas, but still remaining at high level in 2008. The indicator of blood test rate is a newly added indicators in the national health services survey in 2008.

**Infectious diseases:** In addition to routine reporting system on infectious diseases that provides timely information on cases of diseases found from health facilities, national surveys on specific diseases are organized to provide in-depth analysis of the disease control. Using national health services survey, Table S2 presents cure rates of tuberculosis by urban and rural areas. It is noticed that the cure rates declined between 2003 and 2008 in both urban and rural areas with higher cure rate for rural tuberculosis cases.

**General curative care:** A number of indicators have been used to measure access and health care utilization of curative care services, including distance from residence place to health providers, proportions of people who did not seek health care when they were ill, and proportions of people did not use inpatient care when they were advised by doctors to do. The last part in Table S2 presents the proportions of people who did not use inpatient care when they needed by urban and rural areas and by income quintile between 1993 and 2008. In each of the same level income quintile, proportions of unmet inpatient care in rural area were higher than that in urban area. In 2008,71.4% of the people who had unmet inpatient care was due to financial hardship in rural area and 67.5% in urban area, which means financial barrier was still a problem in affecting access to inpatient care even if most of the people had been covered by health insurance schemes at that time.

|  | **1993** | **1998** | **2003** | **2008** |
| --- | --- | --- | --- | --- |
| **Maternal and child health** | | | | |
| Antenatal care (average visits per pregnant woman) |  |  |  |  |
| All | 3.2 | 3.8 | 4.8 | 5.4 |
| Urban | 6.3 | 6.4 | 8.2 | 8.3 |
| Rural | 2.6 | 3.2 | 4.1 | 4.7 |
| Screening of gynecological diseases for those aged between 15-49 years old (%) |  |  |  |  |
| All | - | - | 26.7 | 46.6 |
| Urban | - | - | 36.5 | 56.6 |
| Rural | - | - | 23.2 | 43.3 |
| East | - | - | 31.6 | 54.4 |
| Central | - | - | 26.4 | 43.4 |
| West | - | - | 22.7 | 42.4 |
| Children hepatitis B immunization rate in rural area (%) |  |  |  |  |
| All | - | - | 75.2 | 93.9 |
| Income quintile I (the poorest) | - | - | 57.4 | 90.0 |
| II | - | - | 71.5 | 93.1 |
| III | - | - | 73.2 | 94.9 |
| IV | - | - | 83.5 | 96.0 |
| V (the richest | - | - | 90.4 | 95.6 |
| **Chronic conditions and injuries (CCI)** | | | | |
| Smoking rate (people aged 15 and above years old) (%) |  |  |  |  |
| All | 32.0 | 28.9 | 26.0 | 25.1 |
| Male | 59.3 | 53.4 | 48.9 | 48.0 |
| Female | 5.0 | 4.0 | 3.2 | 2.6 |
| Urban | 30.8 | 27.2 | 23.9 | 22.5 |
| Rural | 32.5 | 29.5 | 26.8 | 26.0 |
| Blood pressure test rate in a year (people aged 35 and above years old) (%) |  |  |  |  |
| All | - | - | - | 49.1 |
| Male | - | - | - | 46.2 |
| Female | - | - | - | 52.0 |
| Urban | - | - | - | 65.3 |
| Rural | - | - | - | 42.4 |
| **Infectious diseases** | | | | |
| Cure rate of tuberculosis (%) |  |  |  |  |
| All | - | - | 96.4 | 89.7 |
| Urban | - | - | 90.5 | 87.4 |
| Rural | - | - | 97.8 | 90.3 |
| **General curative care** | | | | |
| Unmet inpatient care (%) |  |  |  |  |
| All | 30.9 | 31.8 | 29.6 | 25.1 |
| Urban | 23.1 | 27.3 | 27.8 | 26.0 |
| Rural | 34.6 | 34.3 | 30.3 | 24.7 |
| Income quintile I (the poorest) | 43.7 | 42.8 | 32.7 | 31.1 |
| II | 36.3 | 35.9 | 28.1 | 28.0 |
| III | 30.2 | 33.1 | 24.2 | 25.8 |
| IV | 27.0 | 25.7 | 20.1 | 20.8 |
| V (the richest) | 20.4 | 21.1 | 29.6 | 17.6 |

**Table S2:** Coverage of health care services, 1993-2008

Data source: National Health Services Survey, 1993, 1998, 2003, 2008 [15]

**4.1.2 Coverage of services by non-health sector**

Coverage of safe drinking water, hygienic toilet, and use of safe belt are the main health-related indicators reflecting health promotion progress that are contributed from outside health sector. Safe drinking water is measured by use of drinking water provided through a tube system. Figure S3 demonstrates changes in coverage of safe drinking water in rural area by region. From 1987 to 2011, coverage of safe drinking was significantly increased from 19.5% to 72.1%. While gaps in coverage was narrowed between eastern and western regions, it is widened between eastern and central regions. In 2011, there were still about 20% gap in safe drinking water between eastern and other two regions.


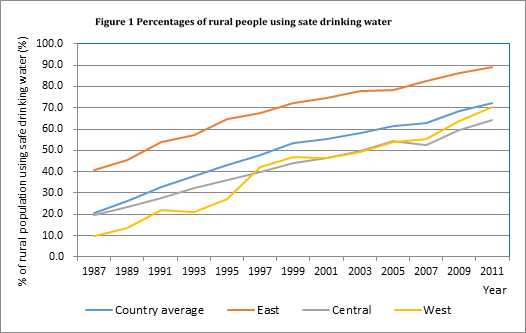


**Figure S3:** Percentage of rural people using safe drinking water

Data source: China Health Statistics Yearbook, 2004-2012 [16]

**4.2 Financial Coverage**

Coverage of health insurance schemes, OOP, and impoverishment rate are used to analyze the progress of financial coverage.

**4.2.1 Population coverage of health insurance schemes**

Medical expenditure is the main source of financial risks for households. To establish prepayment systems in both rural and urban areas has been a key strategy in China to provide financial protections to the people. Figure S1 shows population coverage of the three schemes from 2003 to 2011, showing a rapid expansion from mid 2000s. Rapid expansion of NCMS and the urban resident-based medical insurance (URBMI) in 2007 made significant contribution to a high population coverage, while the urban employee-based medical insurance scheme (UEBMI) has been steadily developed over the past years.

**
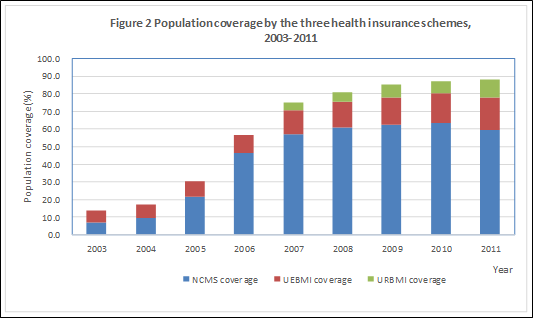
**

**Figure S1:** Population Coverage by the three health insurance schemes, 2003-2011

Data source: China health statistics year book, 2007-2012 [17]; China Statistics 2010-2012 [18]; China Labor Statistics 2010-2011 [19]

**4.2.2 Out-of-pocket payment**

Out-of-pocket payment (OOP) is one of the major indictors used by the central government in measuring achievements of health financing reform and effectiveness of financial protection. Figure S4 illustrates the trends of China’s health expenditures from 1978 to 2011 [20].


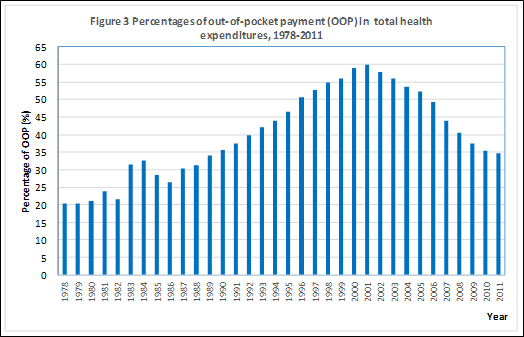


**Figure S4:** Percentages of out-of-pocket payment (OOP) in total health expenditures, 1978-2011

Data source: Report on China’s Health Account Study, 2012 [20]

Between 1978 and 1987, proportions of OOP increased from 20%-30%; between 1988 and 2001, OOP proportions increased from 30% to 60%, reflecting the market-oriented financing in health sector; and OPP proportions started to decline in 2002. It declined to 50% in 2006 and 34.8% in 2011. The rapid declines in recent years are mainly contributed to increases of government investments in health and expansion of health insurance schemes.

**4.2.3 Impoverishment**

The poverty line in this analysis is set at an average food expenditure of households in the 45th to 55th percentiles using the method from WHO [21]. Figure S2 demonstrates percentage of households whose incomes were below the poverty line due to medical expenditures (impoverishment rates) by income quintile in 2003 and 2008. In 2003, the impoverishment rates of households with income quintiles II and III were 10.7% and 9.8%, respectively, which were higher than other income groups. Between 2003 and 2008, the impoverishment rates decreased by 0.9%, but the poorest income group increased dramatically and the second poorest group had no significant change. Impoverishment rates decreased in other income groups.

**
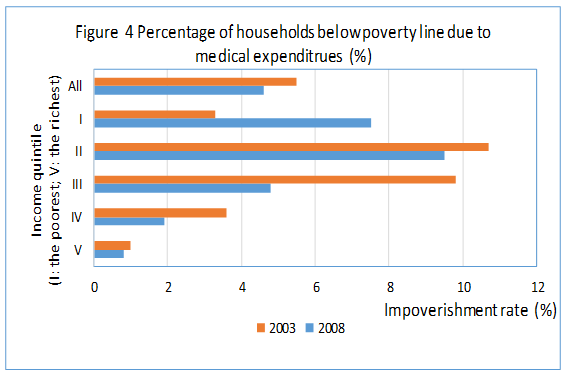
**

**Figure S2:** Percentage of households below poverty line due to medical expenditures (%)

Data source: National health services surveys in 2003 and 2008

**5. Conclusions and recommendations**

Analysis of the progress towards UHC with selected indicators shows positive trends, especially on dimension of service coverage [17]. In financial protection, nearly all people have been covered by either rural health insurance scheme or urban health insurance schemes. Even though impoverishment rate continued to increase for the poor between 2003 and 2008, the overall impoverishment rate and proportion of out-of-pocket in total health expenditures have declined.

A number of challenges need to be addressed for accelerating UHC in China. Firstly, equity in health needs to be further improved. Besides existence of disparities in service coverage between urban and rural area, regions, and population groups, inequity in finance is a big concern. The fund level and benefit package in rural health insurance scheme (NCMS) and urban resident-based health insurance scheme are much lower than the urban employee-based health insurance scheme. A mobile health protection mechanism has not been available for the rural migrants. Secondly, a big space exists for improvements of health care quality. While access to health care has been improved, quality of care has not been significantly advanced mainly due to limited availability of qualified health workers in primary health facilities. In line with increase of benefit package of health insurance schemes, demand for high quality of health care would increase accordingly, which requires more significant progress of quality improvements. Thirdly, cost escalation of medical care should be appropriately contained. After introduction of the health insurance schemes, cost has escalated mainly resulted from the increasing of health care utilization. However, unnecessary care exist due to moral hazards from the insured and induced demand from health providers, which pushes rapid increase in medical cost and negatively affect capacity of financial protection of the financing mechanisms. Finally, the concept of health in all policies needs to be operationalized. UHC cannot be achieved solely relying on health sector. Effective cooperation between related sectors targeting the major problems in health system is critical for UHC.

In the meantime, information systems need to be improved for better monitoring and evaluation of UHC. In both routine report system and surveys, efforts for improving quality of data are needed, including better organization of trainings of the field investigators and monitoring of quality of data collection. Coordination in development and utilization of the indicators of UHC monitoring and evaluation between relevant organizations and institutions needs to be improved. Many of the indicators are related to multiple government agencies, which need agreement from those agencies to define and utilize the indicators. However, it is not easy to coordinate the efforts. In addition, constraints exist for sharing information mainly because databases are usually managed by single organization without a mechanism to encouraging sharing.

Recommendations are proposed as listed in Box S1 for addressing the challenges.

| **Box S1: Recommendations**  1. Gaps in health financing and benefit packages between the three health insurance schemes should be gradually closed through more rapidly increasing of financial support to the rural health insurance scheme. Ability-based premium contribution and cost sharing mechanisms in health financing can be explored to reduce financial burdens of the poor.  2. Primary health care system should be continuously strengthened. Strategies for mobilizing qualified health workers and other resources are needed for primary health organizations to significantly improve quality of care provided, from which access to quality health care by the communities can be improved.  3. Escalation of medical cost should be reasonably controlled. Cost escalation of medical care is a threat to sustainability of health financing and extension of health benefits to the people. To control unnecessary care from the demand side and to improve rational supply of health care through provider payment reforms would be the key strategies.  4. Coordination between sectors for achieving UHC can be improved. An accountability system in which responsibilities of UHC-related sectors are clearly defined needs to be established.  5. Information system for monitoring and evaluation of UHC can be improved. Different sources of databases related to UHC can be coordinated in use to increase availability and reliability of information. Local governments and organizations can be encouraged to carry out information collection and analysis to reflect their local situations in process towards UHC. |
| --- |

**References**

1. National Population Census (2011) Report on the Sixth National Population Census. Beijing.
2. National Population and Family Planning Commission. Department of Migration Management and Services (2013) Report on China’s migrant population development 2012. China Population Publishing House, Beijing.
3. China National Bureau of Statistics (2011) China Statistical Yearbook. Beijing: China Statistical Press.
4. World Bank. <http://publications.worldbank.org/WDI/indicators>, access on October 24, 2013.
5. The National Health and Family Planning Commission (2012) Digest of Health Statistics. Beijing: The National Health and Family Planning Commission.
6. China National Bureau of Statistics (2012) China Statistical Yearbook. Beijing: China Statistical Press.
7. Tang S, Meng Q, Chen L, Bekedam H, et al. (2008) Tackling the challenges to health equity in China. Lancet 372: 1493-1501.
8. Wagstaff A, Lindelow M, Gao J, Xu L, Qian J (2009) Extending health insurance to the rural population: an impact evaluation of China’s New Cooperative Medical Scheme. Journal of Health Economics 28: 1-19.
9. Central Committee of Chines Communist Party and the State Council (2009). Decision on health systems reform. Beijing.
10. World Health Organization (2010) Health Systems Financing: the Path to Universal Coverage, Geneva, WHO Publication
11. Lei H (2012) Measuring universal health coverage in China. Technical Report from a UHC Measurement project. Beijing
12. Meng Q and Tang S (2010). Universal coverage of health care in China: challenges and opportunities. World Health Report Background Paper, No 7. Geneva: WHO Publication
13. Central Committee of Chines Communist Party and the State Council (2002) Strengthening the rural health system. Beijing.
14. The State Council (2006) Guideline for developing urban resident-based basic health insurance scheme. Beijing.
15. Center for Health Statistics and Information, National Health and Family Planning Commission. National Health Services Survey, 1993, 1998, 2003, and 2008.
16. China National Bureau of Statistics (2012). China Statistical Yearbook. Beijing: China Statistical Press.
17. Ministry of Health. China Health Statistics Yearbook (2007-2012). Beijing: China Statistical Press.
18. China National Bureau of Statistics. China Statistical Yearbook (2010-2012). Beijing: China Statistical Press.
19. Ministry of Human Resource and Social Security (2012) China Labor Statistics 2011. Beijing: China Statistical Press.
20. China National Health Development Research Center (2012) China National Health Account Report 2012. Beijing.
21. Xu K, Evans D, Kawabata K, Zeramdini R, et al.（2003）Household catastrophic health expenditure: a multicountry analysis. Lancet 362: 111–17.
